# Coenzyme Q10 Attenuates Human Platelet Aggregation Induced by SARS-CoV-2 Spike Protein via Reducing Oxidative Stress In Vitro

**DOI:** 10.3390/ijms232012345

**Published:** 2022-10-15

**Authors:** Ruijie Wang, Yiting Chen, Zezhong Tian, Meiyan Zhu, Bingying Zhang, Sijin Du, Yanzhang Li, Zhihao Liu, Shanshan Hou, Yan Yang

**Affiliations:** 1 School of Public Health (Shenzhen), Shenzhen Campus of Sun Yat-sen University, Sun Yat-sen University, Shenzhen 518107, China; 2 Guangdong Provincial Key Laboratory of Food, Nutrition and Health, Sun Yat-sen University, Guangzhou 510080, China; 3 Guangdong Engineering Technology Center of Nutrition Transformation, Sun Yat-sen University, Guangzhou 510080, China

**Keywords:** SARS-CoV-2, spike protein, platelet, CoQ10, oxidative stress

## Abstract

Platelet hyperreactivity and oxidative stress are the important causes of thrombotic disorders in patients with COVID-19. Oxidative stress, induced by the excessive generation of reactive oxygen species (ROS), could increase platelet function and the risk of thrombus formation. Coenzyme Q10 (CoQ10), exhibits strong antioxidative activity and anti-platelet effect. However, the effects and mechanisms of CoQ10 on attenuating platelet aggregation induced by spike protein have never been studied. This study aims to investigate whether the SARS-CoV-2 spike protein potentiates human platelet function via ROS signaling and the protective effect of CoQ10 in vitro. Using a series of platelet function assays, we found that spike protein potentiated platelet aggregation and oxidative stress, such as ROS level, mitochondrial membrane potential depolarization, and lipid damage level (MDA and 8-iso-PGF2α) in vitro. Furthermore, CoQ10 attenuated platelet aggregation induced by spike protein. As an anti-platelet mechanism, we showed that CoQ10 significantly decreased the excess production of ROS induced by spike protein. Our findings show that the protective effect of CoQ10 on spike protein-potentiated platelet aggregation is probably associated with its strong antioxidative ability.

## 1. Introduction

Platelet hyperreactivity such as increased platelet aggregation and activation, is one of the important characteristics of COVID-19, manifesting as an increased risk of thrombotic disorders [1,2]. Platelet hyperreactivity in COVID-19 patients may result from multiple reasons, such as SARS-CoV-2 interacting with platelet directly [3]. Oxidative stress plays a crucial role in the pathogenesis of COVID-19 and perpetuates the blood clotting mechanism [4]. Moreover, viral infections induce high oxidative stress, which also plays a pivotal role in platelet hyperactivity [5]. Oxidative stress is defined as the excessive formation and insufficient removal of highly reactive molecules, such as reactive oxygen species (ROS) which serves as an important second messenger for intracellular signaling in platelet and can affect platelet physiology [5,6,7]. But whether SARS-CoV-2 enhances platelet reactivity via ROS signaling is still unclear.

Interestingly, natural antioxidants in food and medicinal plants, exhibit safety and a wide range of biological effects, including antiviral and anti-atherosclerosis [8]. Some evidence indicates that a healthy diet with supplemental antioxidant intake is beneficial to patients with COVID-19 [9]. Therefore, antioxidants supplement from food sources may be a useful strategy for the treatment and prevention of oxidative stress and platelet hyperreactivity in COVID-19 patients.

Coenzyme Q10 (CoQ10), or ubiquinone, a fat-soluble molecule with strong antioxidant activity, has a physiological critical role in mitochondrial bioenergetics [10]. Our previous study demonstrated that CoQ10 could attenuate platelet integrin αⅡbβ3 signaling and thrombus growth [11] and CoQ10 could reduce platelet ROS level stimulated by collagen and thrombin (data not published). However, whether CoQ10 could reduce platelet ROS level and aggregation potentiated by SARS-CoV-2 spike protein in vitro is still unclear.

Our present study aims to investigate whether SARS-CoV-2 spike protein enhances platelet aggregation via upregulating platelet ROS level and the protective effects of CoQ10 in vitro.

## 2. Results

### 2.1. Spike Protein Potentiates Platelet Aggregation

**Figure 1:**
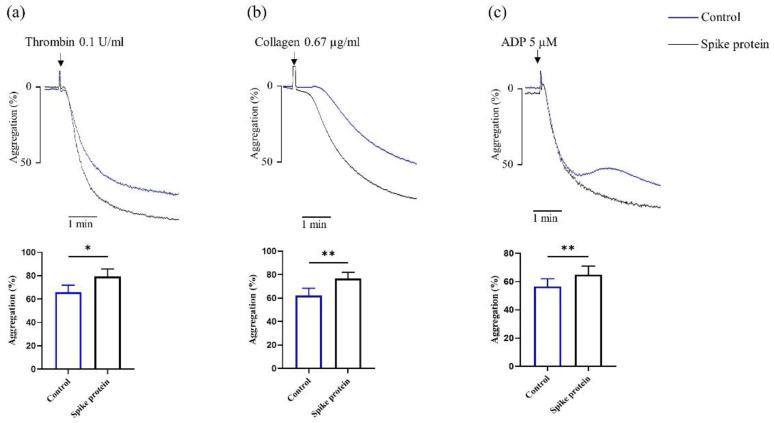
**Spike protein potentiates platelet aggregation.** Platelets were incubated with 2 μg/mL spike protein for 10 min, then gel-filtered platelets activated with (**a**) 0.1 U/mL thrombin (*n* = 4), (**b**) 0.67 μg/mL collagen (*n* = 4), and (**c**) PRP stimulated with 5 μM ADP (*n* = 6), the platelet aggregation was monitored for 5 min. All data are presented as mean ± SEM. * *p* < 0.05, ** *p* < 0.01 indicate a significant difference compared to the control group.

As shown in Figure 1a–c, we found that SARS-CoV-2 spike protein increased platelet aggregation in response to different agonists. These observations collectively suggest that spike protein directly potentiates platelet aggregation in vitro.

### 2.2. Spike Protein Does Not Affect Platelet Activation

**Figure 2:**
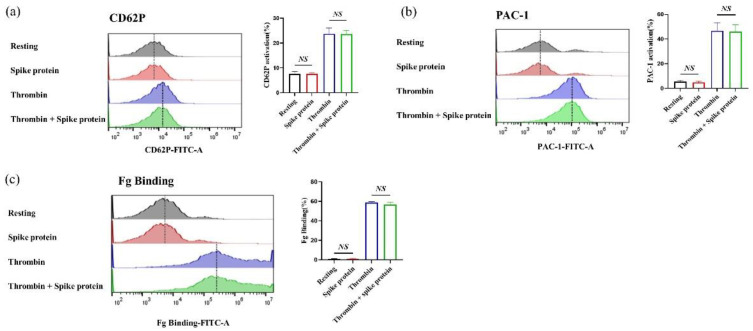
**Spike protein does not affect platelet activation.** Platelets were incubated with spike protein (2 μg/mL, 10 min) in the absence of agonist, or pre-incubated with spike protein at 2 μg/mL for 10 min and stimulated with thrombin (0.1 U/mL) for 10 min, and then the expression of (**a**) CD62P (*n* = 4), (**b**) the PAC-1 binding (*n* = 3), and (**c**) fibrinogen binding (*n* = 3), were analyzed using a flow cytometer. All data are presented as mean ± SEM. *NS*, no significant difference between two groups.

Furthermore, we found that spike protein did not affect the expression of P-selectin and the activation of integrin αⅡbβ3 in the absence or presence of the agonist (Figure 2). These findings demonstrate that spike protein could not induce platelet activation in vitro.

### 2.3. CoQ10 Attenuates Spike Protein-Potentiated Platelet Aggregation

**Figure 3:**
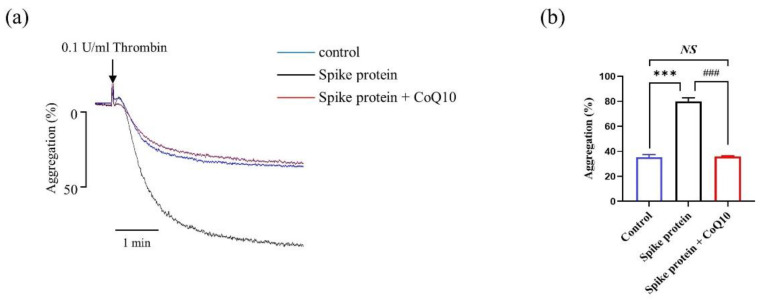
**CoQ10 attenuates spike protein-potentiated platelet aggregation.** Platelets were incubated with 100 μM CoQ10 or solvent for 50 min in the dark, then incubated with 2 μg/mL spike protein for 10 min. The samples were then activated with 0.1 U/mL thrombin, and the platelet aggregation was monitored for 5 min. (**a**) Representative traces and (**b**) summary data were presented (*n* = 3). All data are presented as mean ± SEM. *** *p* < 0.001 indicates a significant difference compared to the control group. ### *p* < 0.001 indicates a significant difference between two groups. *NS*, no significant difference between two groups.

Since spike protein potentiated platelet aggregation in response to thrombin, we observed that CoQ10 significantly attenuated spike protein-potentiated platelet aggregation in vitro (Figure 3).

### 2.4. CoQ10 Inhibits Spike Protein-Potentiated Platelet Oxidative Stress

**Figure 4:**
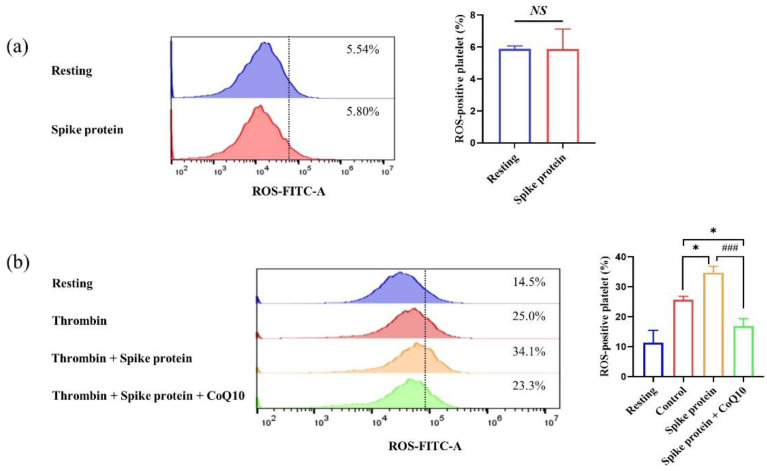
**CoQ10 inhibits spike protein-potentiated platelet intracellular ROS.** (**a**) DCFH-DA incubated platelets were incubated with 2 μg/mL spike protein for 10 min (*n* = 3). (**b**) DCFH-DA incubated platelets were incubated with 100 μM CoQ10 or solvent for 50 min in the dark, then incubated with 2 μg/mL spike protein for 10 min, followed by stimulation with 1 U/mL thrombin for 30 min. ROS-positive platelet was measured by flow cytometry (*n* = 4). All data are presented as mean ± SEM. * *p* < 0.05 indicate a significant difference compared to the control group. ### *p* < 0.001 indicates a significant difference between two groups. *NS*, no significant difference between two groups.

Although previous results have demonstrated that SARS-CoV-2 spike protein induced platelet hyperreactivity, the effect of spike protein on platelet oxidative stress is still unknown. We found that spike protein did not affect resting platelet ROS (Figure 4a). Since we have demonstrated that the interaction between spike protein and integrin β3 was increasing and the outside-in signaling was upregulating after being stimulated with thrombin (data not shown), we explore whether spike protein affects activated platelet oxidative stress. Surprisingly, we observed that 2 μg/mL spike protein increased activated platelet ROS level and this effect was attenuated in the presence of CoQ10 (Figure 4b). 

**Figure 5:**
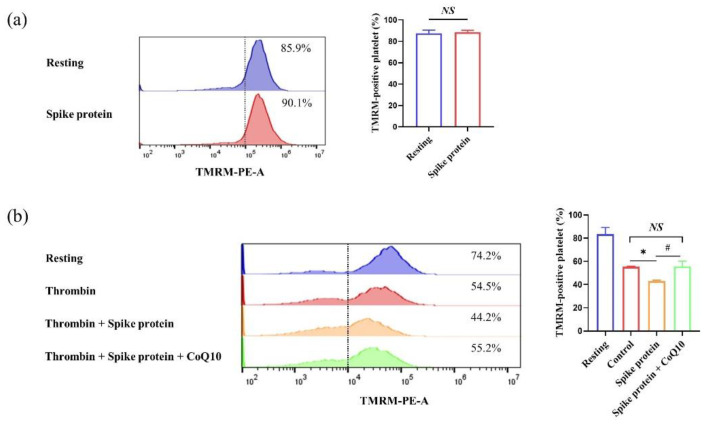
**CoQ10 inhibits spike protein-potentiated platelet mitochondrial membrane potential dissipation.** (**a**) Platelets were incubated with 2 μg/mL spike protein for 10 min, then incubated with 400 nM TMRM for 20 min (*n* = 3). (**b**) Platelets were incubated with 100 μM CoQ10 or solvent for 50 min in the dark, then incubated with 2 μg/mL spike protein for 10 min, followed by stimulation with 1 U/mL thrombin for 30 min, then incubated with 400 nM TMRM for 20 min (*n* = 3). TMRM-positive platelet was measured by flow cytometry. All data are presented as mean ± SEM. * *p* < 0.05 indicates a significant difference compared to the control group. # *p* < 0.05 indicates a significant difference between two groups. *NS*, no significant difference between two groups.

To determine the impact of spike protein on mitochondrial function, we used TMRM staining to measure the mitochondrial membrane potential (ΔΨm). Similarly, we found that spike protein did not affect resting platelet ΔΨm. But spike protein could increase the dissipation of activated platelet ΔΨm, whereas CoQ10 attenuated this effect (Figure 5). Therefore, these results demonstrate that spike protein potentiates activated platelet oxidative stress, and CoQ10 inhibits this spike protein-mediated effect.

### 2.5. CoQ10 Inhibits Platelet Oxidative Lipid Damage Induced by Spike Protein

**Figure 6:**
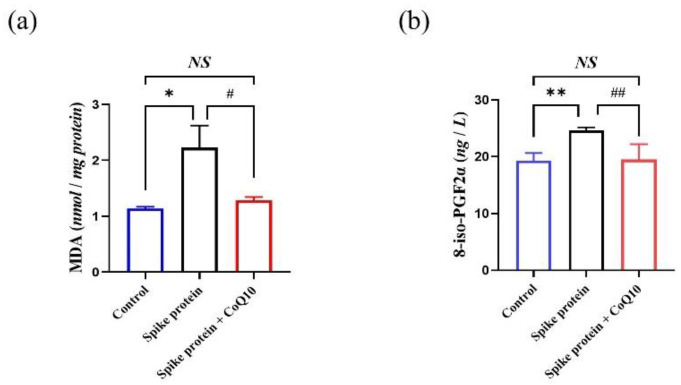
**CoQ10 inhibits platelet oxidative lipid damage induced by spike protein.** Gel-filtered platelets were incubated with CoQ10 or solvent for 50 min, then incubated with spike protein for 10 min, then stimulated with thrombin for 10 min. (**a**) Platelets pellet was lysed for MDA analysis (*n* = 5). (**b**) Supernatant was used for 8-iso-PGF2α level detection (*n* = 4). All data are presented as mean ± SEM. * *p* < 0.05, ** *p* < 0.01 indicate a significant difference compared to the control group. # *p* < 0.05, ## *p* < 0.01 indicate a significant difference between two groups. *NS*, no significant difference between two groups.

The generation of ROS can cause oxidative damage to biological macromolecules. Malondialdehyde (MDA) and 8-iso-prostaglandin-F2α (8-iso-PGF2α) are usually addressed to evaluate oxidative lipid damage which is widely used as markers of oxidative stress in platelet [6,12,13]. We observed that spike protein increased the level of MDA and 8-iso-PGF2α after being stimulated with thrombin, and CoQ10 inhibited these effects (Figure 6a,b).

### 2.6. CoQ10 Improves Platelet Antioxidative Activity Reduced by Spike Protein

**Figure 7:**
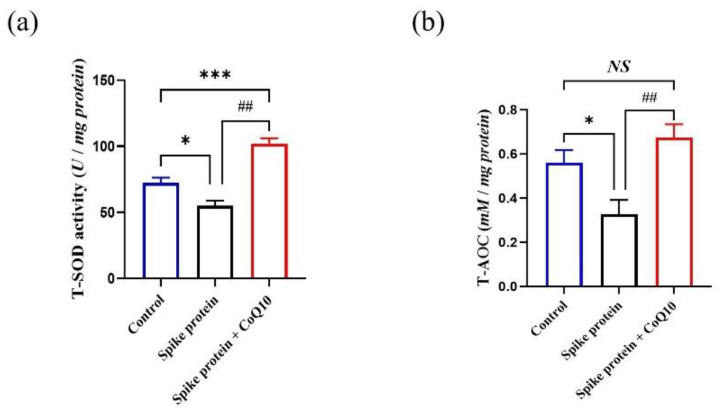
**CoQ10 improves platelet antioxidative activity reduced by spike protein.** Gel-filtered platelets were incubated with CoQ10 or solvent for 50 min, then incubated with spike protein for 10 min, then stimulated with thrombin for 10 min. (**a**) Platelets lysates were used for SOD activity analysis (*n* = 4). (**b**) Platelets lysates were used for T-AOC level measurement (*n* = 5). All data are presented as mean ± SEM. * *p* < 0.05, *** *p* < 0.001 indicate a significant difference compared to the control group. ## *p* < 0.01 indicates a significant difference between two groups. *NS*, no significant difference between two groups.

Moreover, we further detected the antioxidative state in platelets, such as superoxide dismutase (SOD) activity and total-antioxidant capacity (T-AOC) level [14]. We found that CoQ10 could enhance the SOD activity (Figure 7a) and T-AOC level (Figure 7b) which were decreased by spike protein.

### 2.7. The Effect of Spike Protein on Potentiating Platelet Aggregation Is via ROS-Mediated Signaling Pathway

**Figure 8:**
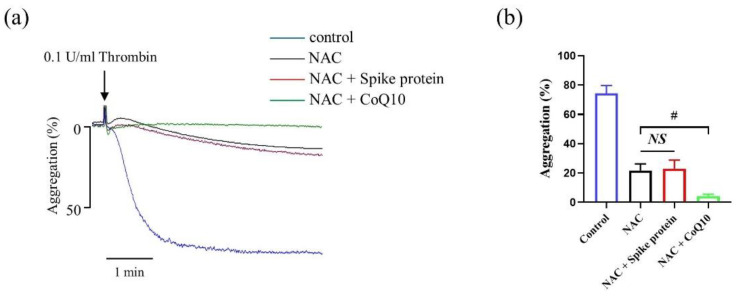
**The effect of Spike protein on potentiating platelet aggregation is via ROS-mediated signaling pathway.** Human gel-filtered platelets were incubated with 1 mM NAC for 20 min, then incubated with spike protein for 10 min or CoQ10 for 50 min. All the samples then were activated with 0.1 U/mL thrombin, and the platelet aggregation was monitored for 5 min. (**a**) Representative traces and (**b**) summary data were presented (*n* = 6). Data are presented as mean ± SEM. # *p* < 0.05 indicates a significant difference between two groups. *NS*, no significant difference between two groups.

As shown in Figure 8, spike protein-mediated increase of platelet aggregation was completely abolished by ROS inhibitor NAC. Moreover, compared with the NAC-mediated effect on platelet aggregation, the combination of NAC and CoQ10 showed further inhibition, indicating that CoQ10 partly attenuated platelet aggregation via reducing ROS level. These data demonstrate that spike protein enhances platelet aggregation by ROS-mediated signaling and CoQ10 could attenuate this effect via eliminating ROS.

## 3. Discussion

Multiple evidence shows that patients with COVID-19 exhibit thromboembolic disorders and platelet hyperreactivity [2]. Platelets play a crucial role in the procession of hemostasis and coagulation [15]. In this article, the protective effect of CoQ10 on spike protein-potentiated platelet aggregation is reported for the first time. Platelet aggregation is the process that platelets adhere to each other at sites of vascular injury and is critical for hemostatic plug and thrombus formation, generally used to evaluate platelet function [16]. However, although we found that spike protein did not affect platelet activation in vitro, platelets were found to be activated in COVID-19 patients [2], indicating indirect reasons like cytokine storm or high oxidative stress [1] rather than spike protein directly induce platelet activation. Consistent with Florian Puhm and colleagues, they found that neither SARS-CoV-2 nor purified spike could activate platelets. They also found that TF activity from SARS-CoV-2-infected cells activated thrombin to induce platelet activation [17], though some studies have reported that spike protein could directly enhance platelet activation [3,18] and these differences may be related to experimental conditions and reagent sources. Moreover, our previous study demonstrated that CoQ10 could attenuate platelet activation after being stimulated with thrombin in vitro [11], suggesting that CoQ10 could protect platelet activation in patients with COVID-19.

**Figure A1:**
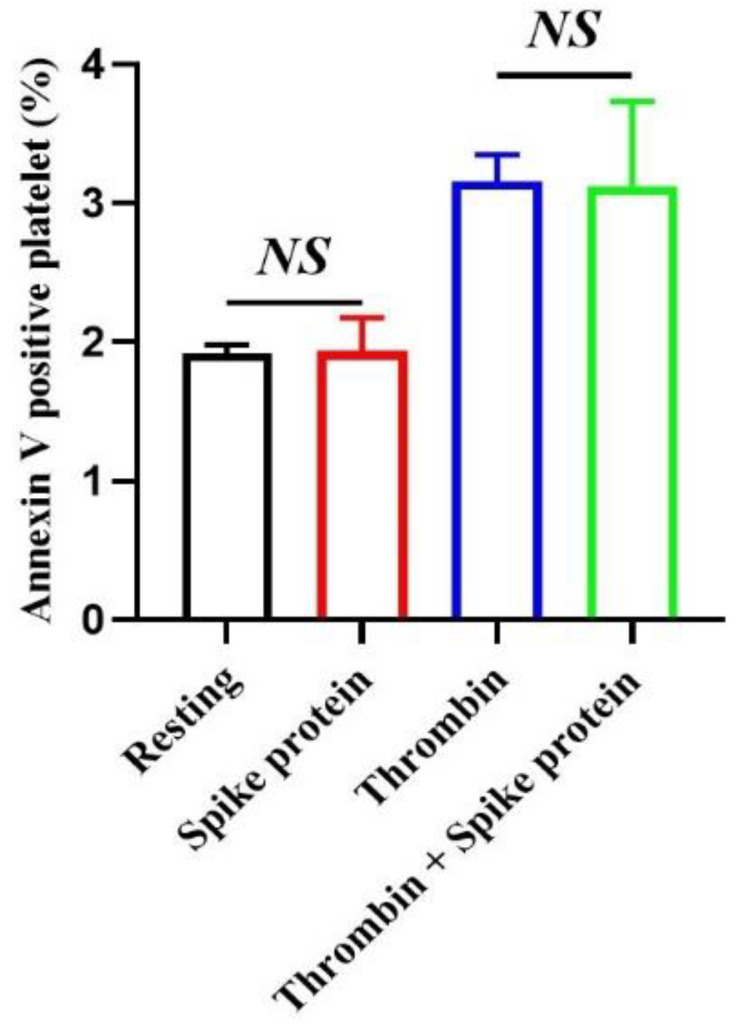
**Spike protein does not affect platelet apoptosis.** Platelets were preincubated with Spike protein at 2 μg/mL for 10 min and stimulated with thrombin (1 U/mL) for 30 min or not, then incubated with Annexin V antibody, platelet apoptosis was assessed by using flow cytometry (*n* = 3). *NS*: no significant difference between two group.

Platelet activation is modulated by extracellular ROS in a microenvironment-dependent manner [19]. After activation, the production of ROS acts as a second messenger to boost platelet activation [6]. The excessive production of ROS could directly alter mitochondrial membrane permeabilization which is associated with subsequent mitochondrial dysfunction [20]. In the present study, we found that only spike protein did not affect resting platelet ROS level and mitochondria function, but after being stimulated with thrombin, spike protein increased activated platelet intracellular ROS level and the depolarization of mitochondria membrane potential. Spike protein has an RGD (arginine-glycine-aspartate) sequence which can bind to integrin αIIbβ3, and our previous study found that after being stimulated with thrombin, since the extracellular domain of αIIbβ3 is changing and increasing ligand binding affinity and avidity [21], the binding of spike protein and αIIbβ3 was increased and outside-in signaling was upregulating (data not shown). These results clarify why spike protein potentiates activated platelet oxidative stress but does not affect resting platelets. Platelets are activated in patients with COVID-19, indicating that SARS-CoV-2 spike protein could upregulate activated platelet ROS level directly to further enhance platelet reactivity. On the other hand, the increased platelet intracellular ROS and mitochondrial dysfunction could induce platelet apoptosis, and exhibit procoagulant activity [22]. However, we found that spike protein did not affect both resting and activated platelet apoptosis (Figure A1), indicating that the mechanism of spike protein-potentiated platelet aggregation was not via platelet apoptosis.

It is reported that platelet mitochondrial function, oxidative phosphorylation, and endogenous CoQ10 level were reduced after COVID-19 [23], indicating that CoQ10 may play a crucial role in platelet function in patients with COVID-19. Interestingly, we observed that CoQ10 could inhibit spike protein-potentiated activated platelets intracellular ROS and the depolarization of mitochondria membrane potential. We also found that in the presence of CoQ10 and spike protein, ROS-positive platelet was even lower than in the control group, and the reason might be that CoQ10 could scavenge the ROS induced by both thrombin and spike protein. Moreover, after pre-incubated with NAC, spike protein-mediated platelet aggregation was eliminated, suggesting spike protein enhanced platelet aggregation via increasing ROS level. Since the combination of NAC and CoQ10 show further inhibition of platelet aggregation, CoQ10 could partly attenuate platelet aggregation due to its antioxidant activity and might also be via the cAMP/PKA pathway partly that was reported previously [11]. Consistent with Diego Méndez, he found that MitoQ, of which the active part is CoQ10, inhibited platelet activation steps via reducing ROS level, and this antiplatelet effect is probably associated with its mitochondrial antioxidant effect [24].

MDA and 8-iso-PGF2α are two classic biomarkers of oxidative stress production. MDA, a principal product of polyunsaturated fatty acid peroxidation, is a highly toxic molecule and is considered a global oxidative stress index [25]. Platelet intracellular ROS activates platelet interaction with arachidonic to give the formation of 8-iso-PGF2α [26], which is reported that has several biologic functions including propagation of platelet activation [27]. Antioxidants may attenuate platelet function indirectly via scavenging of ROS. Antioxidants are important in maintaining redox balance and regulating redox-sensitive signaling pathways in platelets [28]. SOD, one of the antioxidant defenses in platelets, plays an important role in normal platelet function and the prevention of thrombus formation, potentiating endogenous nitric oxide-bioactivity [28]. The measurement of T-AOC is also a reductionist method for evaluating either their radical scavenging or reducing capacity [29]. We found that spike protein increased the production of platelet MDA and 8-iso-PGF2α levels, and decreased the SOD activity and total-antioxidant activity, indicating overproduction of free radicals and defects in the antioxidant system have a crucial role in spike protein-induced platelet hyperreactivity pathogenesis. It is reported that the anti-atherosclerotic mechanism of CoQ10 may be partially due to its antioxidant properties [10]. We further observed that CoQ10 attenuated spike protein-mediated oxidative stress in platelets. Interestingly, Similarly to ROS level, we also found that after pre-incubated with CoQ10 and spike protein, the SOD activity was higher than the control group, indicating that CoQ10 could promote the SOD activity decreased by both spike protein and thrombin. This effect may be attributed to SOD being a potential molecular target of CoQ10. Multiple animal studies, randomized controlled trials and in vitro studies have demonstrated that CoQ10 supplement could improve SOD activity [30,31]. Moreover, plasma oxidative stress markers are significantly elevated in COVID-19 patients, associated with the mechanism of disease development [32]. CoQ10 is also present in blood plasma and could also eliminate excessive circulating ROS in COVID-19 patients, which indirectly inhibits platelet hyperreactivity.

CoQ10 has been used to treat a variety of diseases, especially cardiovascular disease and neurodegenerative diseases [33], and might be a promising treatment for virus infection by its antioxidant ability. Mitochondria play a crucial role in the maintenance of cellular homeostasis. During viral pathogenesis, mitochondrial dynamics are altered by oxidative stress, cytokine production, and so on for the progression of infection [34]. It is reported that CoQ10 levels were significantly lower in patients with COVID-19 or acute influenza infection, and these levels were associated with oxidative stress and inflammatory biomarkers [23,35]. Since CoQ10 acts as a mitochondria electron carrier and a cofactor for mitochondria enzymes, oral CoQ10 treatment is a beneficial strategy during virus infection. A case-control study in a large population that was used to identify the protective drugs to treat COVID-19 found that CoQ10 was associated with reduced hospitalization risk [36]. In addition, Urban Alehagen and colleagues found that dietary supplementation with selenium and CoQ10 could prevent the increase of plasma D-Dimer, an excellent indicator for monitoring thrombosis, which was elevated in COVID-19 [37].

In our previous study, we observed that 100 μM CoQ10 could significantly attenuate platelet function in vitro [11]. Since platelets cannot be intervened too long in vitro, we use a high concentration of CoQ10 (100 μM) for short time intervention to explore the protective effect after being stimulated with spike protein in the present study. It is reported that 1200 mg/day oral dose of CoQ10 supplement in humans was safe [38], and CoQ10 neither shows cytotoxic to platelets nor increases bleeding in mice [11,39], indicating that CoQ10 supplement is safe for COVID-19 patients.

A limitation of the present study is that only the spike protein-potentiated platelet function model was used to explore the protective effect of CoQ10 in vitro. SARS-CoV-2 also needs to use to confirm the effect of CoQ10 in vitro. Moreover, further clinical trials need to confirm whether CoQ10 could attenuate platelet function and oxidative stress in patients with COVID-19.

## 4. Materials and Methods

### 4.1. Materials

SARS-CoV-2 (2019-nCoV) spike protein was purchased from Sino Biological. Coenzyme Q10 (CoQ10), 2′,7′-Dichlorofluorescin diacetate (DCFH-DA), ADP, thrombin from human plasma, and N-acetylcysteine (NAC) were obtained from sigma-Aldrich (St. Louis, MO, USA). Collagen was obtained from Chrono-Log Corp. (Havertown, PA, USA). FITC mouse anti-human CD62P and FITC Mouse Anti-Human PAC-1 were purchased from BD bioscience (San Jose, CA, USA). Alexa Fluor^TM^ 488-conjugated fibrinogen was purchased from Thermo Fisher Scientific (Waltham, MA, USA). TMRM assay kit (mitochondrial membrane potential) was purchased from Abcam (Cambridge, UK). Prostaglandin E1 (PGE1) was obtained from MedChemExpress (Monmouth Junction, NJ, USA). MDA, SOD, and T-AOC assay kits were purchased from Nanjing Jiancheng (Nanjing, China) and the 8-iso-PGF-2α assay kit was obtained from Andy gene (Beijing, China).

### 4.2. Preparation of Human Platelets

Platelets were obtained by phlebotomy from 15 healthy donors who had not taken anti-platelet drugs last three months. This study was reviewed and approved by the Ethics Committee of School of public health (Shenzhen), Sun Yat-Sen University in China [[2020] No. 013] and all healthy donors have signed a written informed consent. Whole blood was obtained with 3.8% sodium citrate (1/9, *v*/*v*) and centrifuged at 1000× rpm for 10 min at 22 °C to collect platelet-rich plasma (PRP). Then, gel-filtered platelets were isolated from the PRP using a Sepharose 2B column according to previously published methods [11].

### 4.3. Platelet Aggregation and Activation Assessment

Platelets (2.5 × 10^8^ platelets per mL) were incubated with 2 μg/mL spike protein for 10 min, then stimulated with different agonists. To explore the effect of CoQ10 on spike protein-potentiated platelet aggregation, gel-filtered platelets were pre-incubated with 100 μM CoQ10 or solvent (0.5% DMSO) in the dark, then incubated with 2 μg/mL spike protein for 10 min and platelet aggregation was stimulated with 0.1 U/mL thrombin. Platelet aggregation was evaluated by an aggregometer (Chrono-Log Corp.). The concentration of CoQ10 is referred to our previous study [11]. For platelet activation assessment, platelets were incubated with FITC-conjugated anti-human CD62P or PAC-1 antibodies, or Alexa Fluor^TM^ 488-conjugated fibrinogen for 20 min at room temperature after pre-treatment. Platelets were then stimulated with 0.1 U/mL thrombin for 10 min or not, and were fixed with 1% paraformaldehyde and then analyzed using flow cytometry (Backman Coulter Inc., Pasadena, CA, USA).

### 4.4. Measurement of Platelet Intracellular ROS

Gel-filtered platelets (1 × 10^7^ platelets per mL) were incubated with 10 μM DCFH-DA for 30 min and centrifuged at 600× *g* for 3 min at 22 °C in the presence of 100 nM PGE1. The platelet pellet was suspended in PIPES buffer to prepare DCFH-DA incubated platelet. DCFH-DA incubated platelets were incubated with 100 μM CoQ10 or solvent for 50 min in the dark. then incubated with 2 μg/mL spike protein for 10 min, followed by stimulation with 1 U/mL thrombin for 30 min. ROS-positive platelet was measured by flow cytometry. 

### 4.5. Measurement of Platelet Mitochondrial Membrane Potential

Mitochondrial membrane potential was determined using the TMRM Assay Kit. In brief, gel-filtered platelets (1 × 10^6^ platelets per mL) were incubated with 100 μM CoQ10 or solvent for 50 min in the dark, then incubated with 2 μg/mL spike protein for 10 min, followed by stimulation with 1 U/mL thrombin for 30 min, then incubated with 400 nM TMRM for 20 min. TMRM-positive platelet was measured by flow cytometry.

### 4.6. Platelet MDA, 8-iso-PGF2α, SOD Activity, and TAC Levels Assay

After incubation with CoQ10 and spike protein, platelets were stimulated with 1 U/mL thrombin for 10 min. Supernatant and cell pellet were collected after centrifugation for 15 min at the speed of 12,000 rpm. Platelets were lysed with lysis buffer. Then MDA, 8-iso-PGF-2α, SOD, and T-AOC assay kits were used to detect their levels in supernatant or platelets lysate. 

### 4.7. Statistical Analysis

All data were presented as mean ± standard error of the mean (SEM) of at least three independent experiments and analyzed using SPSS 25.0. One-way analysis of variance (ANOVA) followed by Tukey’s post hoc test was used for multiple comparisons and a 2-tailed paired student’s *t* test was used to compare the difference between 2 paired groups. *p* values < 0.05 was considered to be statistically significant.

## 5. Conclusions

This study showed that SARS-CoV-2 spike protein potentiated platelet aggregation and oxidative stress. And CoQ10, a natural antioxidant from foods, had an inhibitory effect on spike protein-potentiated platelet aggregation due to its antioxidative activity.

## Data Availability

Data described in the manuscript will be made available upon reasonable request.

## References

[1] Hottz E.D., Azevedo-Quintanilha I.G., Palhinha L., Teixeira L., Barreto E.A., Pao C.R.R., Righy C., Franco S., Souza T.M.L., Kurtz P. (2020). Platelet activation and platelet-monocyte aggregate formation trigger tissue factor expression in patients with severe COVID-19. Blood.

[2] Manne B.K., Denorme F., Middleton E.A., Portier I., Rowley J.W., Stubben C., Petrey A.C., Tolley N.D., Guo L., Cody M. (2020). Platelet gene expression and function in patients with COVID-19. Blood.

[3] Li T., Yang Y., Li Y., Wang Z., Ma F., Luo R., Xu X., Zhou G., Wang J., Niu J. (2022). Platelets mediate inflammatory monocyte activation by SARS-CoV-2 spike protein. J. Clin. Investig..

[4] Cecchini R., Cecchini A.L. (2020). SARS-CoV-2 infection pathogenesis is related to oxidative stress as a response to aggression. Med. Hypotheses.

[5] El Haouari M. (2019). Platelet Oxidative Stress and its Relationship with Cardiovascular Diseases in Type 2 Diabetes Mellitus Patients. Curr. Med. Chem..

[6] Masselli E., Pozzi G., Vaccarezza M., Mirandola P., Galli D., Vitale M., Carubbi C., Gobbi G. (2020). ROS in Platelet Biology: Functional Aspects and Methodological Insights. Int. J. Mol. Sci..

[7] Pietraforte D., Vona R., Marchesi A., de Jacobis I.T., Villani A., Del Principe D., Straface E. (2014). Redox control of platelet functions in physiology and pathophysiology. Antioxid. Redox Signal..

[8] Xu D.P., Li Y., Meng X., Zhou T., Zhou Y., Zheng J., Zhang J.J., Li H.B. (2017). Natural Antioxidants in Foods and Medicinal Plants: Extraction, Assessment and Resources. Int. J. Mol. Sci..

[9] Trujillo-Mayol I., Guerra-Valle M., Casas-Forero N., Sobral M.M.C., Viegas O., Alarcon-Enos J., Ferreira I.M., Pinho O. (2021). Western Dietary Pattern Antioxidant Intakes and Oxidative Stress: Importance During the SARS-CoV-2/COVID-19 Pandemic. Adv. Nutr..

[10] Arenas-Jal M., Sune-Negre J.M., Garcia-Montoya E. (2020). Coenzyme Q10 supplementation: Efficacy, safety, and formulation challenges. Compr. Rev. Food Sci. Food Saf..

[11] Ya F., Xu X.R., Shi Y., Gallant R.C., Song F., Zuo X., Zhao Y., Tian Z., Zhang C., Xu X. (2019). Coenzyme Q10 Upregulates Platelet cAMP/PKA Pathway and Attenuates Integrin alphaIIbbeta3 Signaling and Thrombus Growth. Mol. Nutr. Food Res..

[12] Walter M.F., Jacob R.F., Jeffers B., Ghadanfar M.M., Preston G.M., Buch J., Mason R.P. (2004). Serum levels of thiobarbituric acid reactive substances predict cardiovascular events in patients with stable coronary artery disease: A longitudinal analysis of the PREVENT study. J. Am. Coll. Cardiol..

[13] Xuan Y., Gao X., Holleczek B., Brenner H., Schottker B. (2018). Prediction of myocardial infarction, stroke and cardiovascular mortality with urinary biomarkers of oxidative stress: Results from a large cohort study. Int. J. Cardiol..

[14] Meng Y.Y., Trachtenburg J., Ryan U.S., Abendschein D.R. (1995). Potentiation of endogenous nitric oxide with superoxide dismutase inhibits platelet-mediated thrombosis in injured and stenotic arteries. J. Am. Coll. Cardiol..

[15] Semple J.W., Italiano J.E., Freedman J. (2011). Platelets and the immune continuum. Nat. Rev. Immunol..

[16] Jackson S.P. (2007). The growing complexity of platelet aggregation. Blood.

[17] Puhm F., Allaeys I., Lacasse E., Dubuc I., Galipeau Y., Zaid Y., Khalki L., Belleannee C., Durocher Y., Brisson A.R. (2022). Platelet activation by SARS-CoV-2 implicates the release of active tissue factor by infected cells. Blood Adv..

[18] Zhang S., Liu Y., Wang X., Yang L., Li H., Wang Y., Liu M., Zhao X., Xie Y., Yang Y. (2020). SARS-CoV-2 binds platelet ACE2 to enhance thrombosis in COVID-19. J. Hematol. Oncol..

[19] Chen S., Su Y., Wang J. (2013). ROS-mediated platelet generation: A microenvironment-dependent manner for megakaryocyte proliferation, differentiation, and maturation. Cell Death Dis..

[20] Lopez J.J., Salido G.M., Gomez-Arteta E., Rosado J.A., Pariente J.A. (2007). Thrombin induces apoptotic events through the generation of reactive oxygen species in human platelets. J. Thromb. Haemost..

[21] Huang J., Li X., Shi X., Zhu M., Wang J., Huang S., Huang X., Wang H., Li L., Deng H. (2019). Platelet integrin alphaIIbbeta3: Signal transduction, regulation, and its therapeutic targeting. J. Hematol. Oncol..

[22] Schoenwaelder S.M., Yuan Y., Josefsson E.C., White M.J., Yao Y., Mason K.D., O’Reilly L.A., Henley K.J., Ono A., Hsiao S. (2009). Two distinct pathways regulate platelet phosphatidylserine exposure and procoagulant function. Blood.

[23] Sumbalova Z., Kucharska J., Palacka P., Rausova Z., Langsjoen P.H., Langsjoen A.M., Gvozdjakova A. (2022). Platelet mitochondrial function and endogenous coenzyme Q10 levels are reduced in patients after COVID-19. Bratisl. Lek. Listy.

[24] Mendez D., Arauna D., Fuentes F., Araya-Maturana R., Palomo I., Alarcon M., Sebastian D., Zorzano A., Fuentes E. (2020). Mitoquinone (MitoQ) Inhibits Platelet Activation Steps by Reducing ROS Levels. Int. J. Mol. Sci..

[25] Del Rio D., Stewart A.J., Pellegrini N. (2005). A review of recent studies on malondialdehyde as toxic molecule and biological marker of oxidative stress. Nutr. Metab. Cardiovas..

[26] Violi F., Pignatelli P. (2014). Platelet NOX, a novel target for anti-thrombotic treatment. Thromb. Haemost..

[27] Pignatelli P., Carnevale R., Di Santo S., Bartimoccia S., Sanguigni V., Lenti L., Finocchi A., Mendolicchio L., Soresina A.R., Plebani A. (2011). Inherited human gp91phox deficiency is associated with impaired isoprostane formation and platelet dysfunction. Arterioscler. Thromb. Vasc. Biol..

[28] Freedman J.E. (2008). Oxidative stress and platelets. Arterioscler. Thromb. Vasc. Biol..

[29] Marrocco I., Altieri F., Peluso I. (2017). Measurement and Clinical Significance of Biomarkers of Oxidative Stress in Humans. Oxid. Med. Cell Longev..

[30] Zhang Y.L., Huang X.Y., Liu N., Liu M.M., Sun C.R., Qi B.Y., Sun K., Wei X., Ma Y., Zhu L.G. (2022). Discovering the Potential Value of Coenzyme Q10 in Oxidative Stress: Enlightenment From a Synthesis of Clinical Evidence Based on Various Population. Front. Pharmacol..

[31] Xie T., Wang C., Jin Y., Meng Q., Liu Q., Wu J., Sun H. (2020). CoenzymeQ10-Induced Activation of AMPK-YAP-OPA1 Pathway Alleviates Atherosclerosis by Improving Mitochondrial Function, Inhibiting Oxidative Stress and Promoting Energy Metabolism. Front. Pharmacol..

[32] Mehri F., Rahbar A.H., Ghane E.T., Souri B., Esfahani M. (2021). Changes in oxidative markers in COVID-19 patients. Arch. Med. Res..

[33] Zhang Z.W., Xu X.C., Liu T., Yuan S. (2016). Mitochondrion-Permeable Antioxidants to Treat ROS-Burst-Mediated Acute Diseases. Oxid. Med. Cell Longev..

[34] de Las Heras N., Martin Gimenez V.M., Ferder L., Manucha W., Lahera V. (2020). Implications of Oxidative Stress and Potential Role of Mitochondrial Dysfunction in COVID-19: Therapeutic Effects of Vitamin D. Antioxidants.

[35] Chase M., Cocchi M.N., Liu X., Andersen L.W., Holmberg M.J., Donnino M.W. (2019). Coenzyme Q10 in acute influenza. Influenza Other Respir. Viruses.

[36] Israel A., Schaffer A.A., Cicurel A., Cheng K., Sinha S., Schiff E., Feldhamer I., Tal A., Lavie G., Ruppin E. (2021). Identification of drugs associated with reduced severity of COVID-19—A case-control study in a large population. Elife.

[37] Mei H., Luo L., Hu Y. (2020). Thrombocytopenia and thrombosis in hospitalized patients with COVID-19. J. Hematol. Oncol..

[38] Hathcock J.N., Shao A. (2006). Risk assessment for coenzyme Q10 (Ubiquinone). Regul. Toxicol. Pharmacol..

[39] Ya F., Xu X.R., Tian Z., Gallant R.C., Song F., Shi Y., Wu Y., Wan J., Zhao Y., Adili R. (2020). Coenzyme Q10 attenuates platelet integrin alphaIIbbeta3 signaling and platelet hyper-reactivity in ApoE-deficient mice. Food Funct..

